# The temporal trend of placebo response in migraine prevention from 1990 to 2021: a systematic literature review and meta-analysis with regression

**DOI:** 10.1186/s10194-023-01587-0

**Published:** 2023-05-16

**Authors:** Stewart J. Tepper, Jessica Cirillo, Edward Kim, Gil L’Italien, Julie M. Tweedie, Kunal Lodaya, Dushon Riley, Farah Pathan, Nicholas Antaki, Brian H. Nathanson, Peter McAllister

**Affiliations:** 1 Geisel School of Medicine at Dartmouth, Dartmouth Headache Clinic, Hanover, NH USA; 2 Biohaven Pharmaceuticals, New Haven, CT USA; 3 Boston Strategic Partners Inc, 4 Wellington St., Suite 3, Boston, MA USA; 4 OptiStatim, LLC, PO Box 60844, Longmeadow, MA USA; 5 New England Institute for Neurology and Headache, Stamford, CT USA

**Keywords:** Migraine, Placebo response, Preventive treatment, Temporal trend, Meta-analysis, Systematic literature review

## Abstract

**Background:**

Migraine affects 1.1 billion people globally and is the second leading cause of disability worldwide. In clinical trials, treatment efficacy is evaluated by comparing the differential responses in the treatment and placebo arms. Although placebo response in preventive migraine trials has been studied, there is limited research examining temporal trends. This study evaluates the trend of placebo response over thirty years in migraine prevention trials and investigates the association of potential confounders, such as patient, treatment, and study characteristics on placebo response using meta-analysis with regression.

**Methods:**

We conducted literature searches from January 1990 to August 2021 in bibliographical databases (PubMed, Cochrane Library, and EMBASE). Studies were selected according to PICOS criteria and included randomized, double-blind, placebo-controlled trials evaluating preventive migraine treatments in adult patients diagnosed with episodic or chronic migraine, with or without aura. The protocol was registered with PROSPERO (CRD42021271732). Migraine efficacy outcomes included were either continuous (e.g., monthly migraine days) or dichotomous (e.g., ≥ 50% responder rate (yes/no)). We assessed the correlation of the change in outcome from baseline in the placebo arm, with the year of publication. The relationship between placebo response and year of publication was also assessed after accounting to confounders.

**Results:**

A total of 907 studies were identified, and 83 were found eligible. For the continuous outcomes, the change from baseline in mean placebo response showed an increase over the years (rho = 0.32, *p* = 0.006). The multivariable regression analysis also showed an overall increase in placebo response over the years. The correlation analysis of dichotomous responses showed no significant linear trend between publication year and mean placebo response (rho = 0.08, *p* = 0.596). Placebo response also varied by route of administration.

**Conclusion:**

Placebo response increased over the past 30 years in migraine preventive trials. This phenomenon should be considered when designing clinical trials and conducting meta-analyses.

**Supplementary Information:**

The online version contains supplementary material available at 10.1186/s10194-023-01587-0.

## Background

Migraine affects approximately 1.1 billion people globally and is the second leading cause of disability worldwide [1]. Migraine is a debilitating neurological condition affecting 18% of the individuals in the United States [2, 3]. Treatments evolved over time, from the use of beta-blockers and amitriptyline to the recent emergence of anti-calcitonin gene-related peptide (CGRP) therapies as both acute and/or preventive treatments [4–6]. In randomized controlled trials (RCTs), treatment efficacy is evaluated by comparing the differential responses of the treatment and placebo arms (specific treatment effect). “Placebo response” in analgesia refers to improvement in pain symptoms from a psychological effect, and consists of all factors related to analgesia in the placebo arm of a clinical trial, which are initiated and maintained by expectations of symptom change and changes in emotions or motivation of a patient. Several types of placebo responses are driven by different mechanisms depending on the specific context the placebo is administered, such as route of administration [7, 8]. Moreover, recent failures of clinical trials of analgesics for treating neuropathic pain symptoms have led to speculation about the underlying reasons, and increasing placebo responses is one of them [9]. Placebo response have increased considerably over the years, but drug responses have remained stable, leading to decreased treatment effect.

Research indicates increasing placebo response and decreasing treatment effect size over the years in neuropathic pain and schizophrenia [8, 10]. Since most trials for neurological conditions use subjective patient-reported outcomes, they are especially sensitive to placebo response [11, 12]. Degree of response to placebo in migraine studies shows a significant association with the percent of placebo responders [13], and the route of administration and type of treatment affect placebo response. Other studies reported placebo response for specific migraine treatments such as CGRP antibodies. Forbes et al., evaluated contextual treatment effects of CGRP antibodies in adults with episodic migraine (EM) and chronic migraine (CM), and found that approximately 66% of the reduction in migraine days is due to contextual or placebo effects. However, there are limited comprehensive literature reviews and meta-analyses (MAs) that specifically examined placebo response over years in migraine prevention trials, especially since efficacy outcomes reported in migraine RCT evolved over years, and these changes may have affected the temporal trends in placebo response [13, 14]. Additionally, previous MAs did not adequately assess potential confounders affecting placebo response in all migraine types [13, 14]. Addressing these gaps may improve design of clinical trials and affect clinical practice. This study aims to evaluate placebo response over the years in migraine prevention, and examine the association of potential confounders on placebo response to address this knowledge gap using a meta-analysis with regression.

## Methods

### Literature search

We conducted a systematic comprehensive literature review from January 1990 to August 2021 in three bibliographical databases: PubMed (08/15/2021), Cochrane Library (08/13/2021), and EMBASE (08/06/2021), using a combination of search terms indicating trial design, migraine, prophylaxis treatment, and placebo (Additional files 1 and 2). Search keywords included “randomized controlled trials”, “placebo”, “migraine”, “prevention” and their derivatives. To align with the International Classification of Headache Disorders (ICHD) first published in 1988 (now known as the ICHD-1), we chose to assess placebo response from 1990 to 2021. PubMed, Cochrane Library, and EMBASE index peer-reviewed literature and are amongst the top three research databases with special focus on healthcare and medicine. These are the most widely used and contain the highest number of published articles as well, hence our choice of databases for the review. We also reviewed the studies cited in two previously published meta-analyses and comprehensive reviews evaluating placebo response in migraine prophylaxis trials (Meissner, 2013; Evans, 2021) [13, 15]. The study protocol was registered with the International Prospective Register of Systematic Review Protocols (PROSPERO), protocol registration number CRD42021271732.

### Eligibility criteria and study selection

Filters were applied to limit studies published in English between 1990 and 2021 and, where possible, by trials (Cochrane Library). Studies were selected according to PICOS criteria: population (adults 18 years or older diagnosed with episodic or chronic migraine, diagnosed according to International Classification of Headache Disorders criteria or ICHD criteria), intervention (prophylactic migraine treatments administered orally or via intravenous (IV), intramuscular, or subcutaneous injection), comparison (patients in the intervention group treated with the active drug for migraine prevention; patients in the control group treated with placebo), outcomes (migraine-related efficacy outcomes), and study (randomized, double-blinded, placebo-controlled trials) [16–18]. Studies were required to report at least one migraine-related preventive efficacy outcome. Studies were excluded if they included children or adolescents (< 18 years of age) or had mixed population results (such as adolescents and adults), or included patients diagnosed with menstrual migraine, trigeminal autonomic cephalalgias, or non-migraine headache conditions. Studies that reported acute migraine treatment, homeopathic remedies, or preventive treatments other than oral or injectables (such as acupuncture or sham surgery or non-pharmacological treatments) were excluded. Open-label trials, reviews, observational studies, case series and reports, unpublished studies, non-peer-reviewed studies, and crossover studies (except when the results of the first administration were given separately) were also excluded.

Articles identified in the systematic literature search underwent title and abstract screening by two independent reviewers (FP and NA). The articles included after abstract screening were subject to a full-text review for a second round of detailed evaluation for eligibility and removal of duplicates. Disagreements were resolved through discussion with the entire team of authors or with the intervention of a third reviewer (DR).

### Data collection

Relevant data from the remaining eligible full-text articles, including but not limited to study/participant information, treatment characteristics and outcomes, were extracted by two independent reviewers. Data were collected in an Excel database, and then entered independently in Stata/MP 15.1 for Windows (StataCorp LLC, College Station, TX).

This study evaluated eight continuous and eight dichotomous migraine efficacy outcomes. The following continuous outcomes were included by order of preference: monthly migraine days (MMDs), migraine headache days (MHDs) per month, attacks per month, episodes per month, migraine days, attack frequency, headache days per month, and headache days. For dichotomous outcomes, we included the following by order of preference: proportion of patients with a 50% or more decrease in MMDs, MHDs, attack frequency, migraine episodes per month, migraine days, headache attack frequency, headache days, and intensity or duration.

The revised Cochrane risk-of-bias tool (RoB 2) [19] for randomized trials was used by two independent reviewers (FP and NA) to assess risk of bias. The reviewers ranked each bias as “low risk”, “some concerns”, or “high risk”. Disagreements were resolved by discussion among reviewers and, if needed, with the intervention of a third reviewer (DR). The Jadad scale was also used to assess the quality of the randomized controlled trials [20]. The reviewers ranked each study as “low” or “high quality”. This study was exempt from institutional review or ethics approval since it is a systematic literature review and meta-analysis, and no individual patients were identified nor was patient consent necessary. We adhered to the Preferred Reporting Items for Systematic Reviews and Meta-Analyses (PRISMA) checklist and guidelines for systematic reviews and meta-analyses, when possible, to report results [21, 22].

### Data synthesis and analysis

Data analysis methods were established based on feasibility post data extraction. We measured the correlation between the change in outcome from baseline in the placebo arm and year of publication using Spearman’s Rho, such that an increase in placebo response over the years had a positive correlation. Spearman’s Rho was recalculated based on the route of administration as a sensitivity analysis. Mean differences in the change in the placebo arms from baseline were calculated by route of administration (oral, injection, intravenous) and the differences between the three groups were evaluated with a one-way analysis of variance (ANOVA). We summarized the characteristics of the studies using medians and interquartile [25th and 75th percentile] ranges for continuous variables and counts and frequencies for categorical variables. When inference tests were conducted, all observations were considered to be independent and identically distributed. When an inference test assumed normality (e.g., the one-way ANOVA), we confirmed normality visually by inspecting histograms. *P*-values < 0.05 were considered statistically significant and all inference tests were two-tailed.

The relationship between placebo response and year of publication in the placebo arm was also assessed using linear regression with analytic weights based on the sample size of each study (i.e., the weights are inversely proportional to the variance of each study’s mean change in placebo response from baseline) [23]. The relationship between year of publication and placebo response was allowed to be non-linear by using restricted cubic splines (if necessary), while adjusting for the mean age, percent female, route of administration, Cochrane RoB, and other study characteristics. A restricted cubic spline models the relationship between year and placebo response as a curve rather than a straight line and each turning point in the curve is called a knot. Knot locations were chosen by percentiles recommended by Harrell et al. [24]. Both a parse model and a model with all potential confounders were derived. In four studies, the percent female in the placebo arm was not reported but the overall percent female was (Aurora 2007, Monfared 2017, Relja 2007, Smith 2020) [25–28]. Similarly, in 4 studies the mean age for the placebo arm was not reported but the overall mean age was reported (Freitag 2008, Monfared 2017, Smith 2020, Steiner 1998) [26, 28–30]. In these cases, we imputed the overall percent female or overall mean age to be the respective placebo arms’ values instead of remove the studies from the regression analysis. No other imputation was conducted. All analyses were performed in Stata/MP 15.1 for Windows (StataCorp LLC, College Station, TX).

## Results

### Search results

**Fig. 1:**
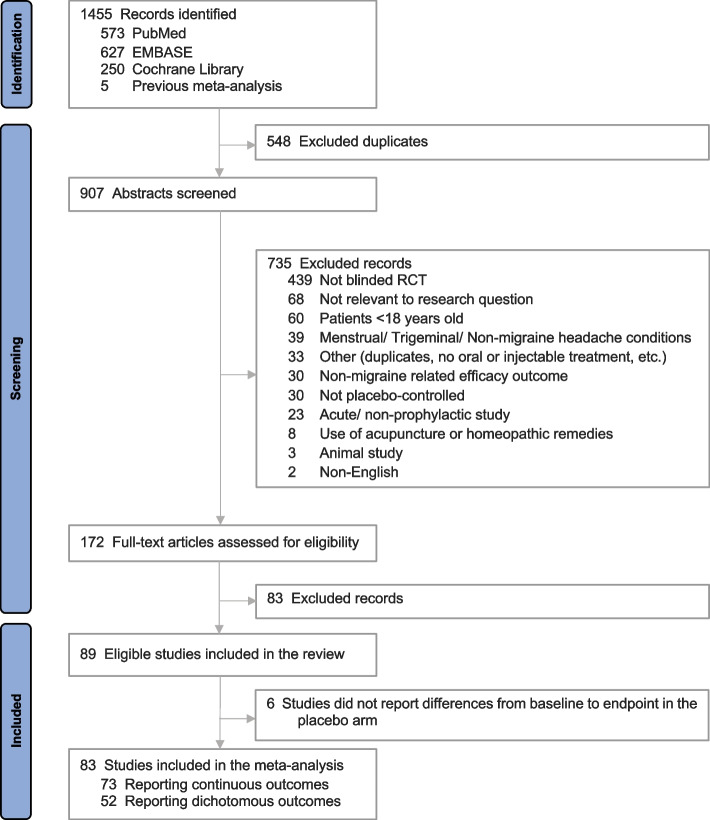
Final cohort of studies included in the meta-analysis (PRISMA Diagram)

The initial literature search returned 1,455 records. Following removal of duplicates, 907 abstracts were screened by two independent reviewers. Of these, 172 articles were considered relevant for full text review. After the full-text review, 83 articles were excluded (reasons for study exclusion are outlined in the PRISMA flow diagram in Fig. 1). From the 89 studies meeting the PICOS inclusion and exclusion criteria, 83 studies containing at least one preventive outcome were included in this study.

### Characteristics of included studies and participants

Out of the 83 studies, continuous and dichotomous data were retrieved from 73 and 52 studies, respectively (one publication [31] reported two separate dichotomous studies). In one study [32], the placebo response was calculated as median and this was transformed to a mean using the method proposed by Hozo [33]. There was an overlap of 43 studies between the two outcomes, 30 studies reported only continuous outcomes, and 10 studies reported only dichotomous outcomes. Study characteristics are shown in Table 1. Studies were published in English between 1996 to 2021, and many were conducted outside the United States. Attack frequency and attacks per month were the more common efficacy measures in the 1990s, whereas, MMDs and MHDs are presently the most common measures in migraine prevention trials. We found that two studies had low quality according to the JADAD scale, and 28 had some concerns or a high risk of bias according to the RoB 2 tool.

**Table 1:** Summary of all included studies (*n* = 83)

Study title	Year	Intervention	Route of Administration	Cochrane Risk of Bias	Outcome Types	Outcome Measure
Abdolahi et al. [34]	2019	ω-3 fatty acids and nano-curcumin	Oral	Low	Continuous	Attack frequency
Anand et al. [35]	2006	Botox A	Injection	Low	Continuous	Attacks per month
Ashina et al. [36]	2020	Eptinezumab	IV	Low	Continuous, Dichotomous	MMDs
Aurora et al. [25]	2007	Botox A	Injection	Some concerns	Continuous	Episodes per month
Aurora et al. [37]	2010	Botox A	Injection	Low	Continuous	MMDs
Barrientos & Chana et al. [38]	2003	Botox A	Injection	Low	Continuous	Attacks per month
Bigal et al. [39]	2015	Fremanezumab	Injection	Low	Continuous	Headache days
Bigal et al. [40]	2015	Fremanezumab	Injection	Low	Continuous	Migraine days
Brandes et al. [41]	2004	Montelukast	Oral	Low	Continuous, Dichotomous	MMDs, Attack frequency
Buettner et al. [42]	2015	Simvastatin plus vitamin D3	Oral	Some Concerns	Continuous, Dichotomous	Migraine days
Cady et al. [43]	2009	Carisbamate	Oral	Low	Dichotomous	MMDs
Casas et al. [44]	2019	DAO supplement	Oral	Some concerns	Continuous	Attacks per month
Chankrachang et al. [45]	2011	Botox A	Injection	Low	Continuous	Attacks per month
Croop et al.[46]	2021	Rimegepant	Oral	Low	Continuous, Dichotomous	MMDs
Detke et al. [47]	2018	Galcanezumab	Injection	Low	Continuous, Dichotomous	MHDs
Diener et al. [48]	2001	Cyclandelate	Oral	Low	Continuous	MMDs
Diener et al. [49]	2005	MIG-99	Oral	Some concerns	Continuous, Dichotomous	Attacks per month, MMDs
Diener et al. [50]	2008	Telmisartan	Oral	High	Continuous, Dichotomous	MMDs
Diener et al. [51]	2010	Botox A	Injection	Low	Continuous	Migraine days
Diener et al. [52]	2007	Topiramate	Oral	Low	Continuous, Dichotomous	MMDs
Diener et al. [53]	1996	Cyclandelate	Oral	Some concerns	Dichotomous	Attack frequency
Dodick et al. [54]	2018	Fremanezumab	Injection	Low	Continuous, Dichotomous	MMDs
Dodick et al. [55]	2014	Eptinezumab	IV	Low	Continuous, Dichotomous	Migraine days, MHDs
Dodick et al. [56]	2018	Erenumab	Injection	Low	Continuous	MMDs
Dodick et al. [57]	2014	Galcanezumab	Injection	Low	Continuous Dichotomous	MHDs, Migraine days
Dodick et al. [58]	2019	Eptinezumab	IV	Low	Dichotomous	MMDs
Elkind et al. [59]	2006	Botox A	Injection	Some concerns	Continuous	Migraine days
Evers et al. [60]	2004	Botox A	Injection	Low	Dichotomous	MMDs
Ferrari et al. [61]	2019	Fremanezumab	Injection	Low	Continuous, Dichotomous	MMDs
Freitag et al. [29]	2008	Botox A	Injection	Low	Continuous, Dichotomous	Episodes per month, MMDs
Fu et al. [62]	2012	Chinese herbal medicine (CXDT-HFG)	Oral	Some concerns	Continuous, Dichotomous	Migraine days
Gaul et al. [63]	2015	Vitamin B2, Magnesium, Coenzyme Q10	Oral	Low	Continuous	MMDs
Goadsby et al. [64]	2017	Erenumab	Injection	Low	Continuous, Dichotomous	MMDs
Goadsby et al. [65]	2009	Tonabersat	Oral	Some concerns	Continuous, Dichotomous	MMDs, MHDs
Goadsby et al. [66]	2020	Atogepant	Oral	Low	Continuous, Dichotomous	MMDs
Gonçalves et al. [67]	2016	Melatonin, Amitriptyline	Oral	Low	Continuous, Dichotomous	MHDs
Hajihashemi et al. [68]	2019	CoQ10 and L-carnitine	Oral	Some concerns	Continuous	Attack frequency
Høivik et al.^b^ [31]	2010	GW274150	*NA*	Low	Dichotomous	MHDs
Kisler et al. [69]	2019	Duloxetine	Oral	High	Continuous	MMDs
Köseoglu et al. [70]	2008	Magnesium	Oral	High	Continuous	Attacks per month
Lipton et al. [71]	2011	Topiramate	Oral	Low	Continuous	MMDs
Lipton et al. [72]	2020	Eptinezumab	IV	Low	Continuous, Dichotomous	MMDs
Lipton et al. [73]	2004	Petasites extract	Oral	Low	Dichotomous	Attack frequency
Magis et al. [74]	2007	Thioctic acid	Oral	Low	Continuous, Dichotomous	Attacks per month, Attack frequency
Mei et al. [75]	2004	Topiramate	Oral	High	Continuous, Dichotomous	MHDs, MMDs
Millan-Guerrero et al. [76]	2006	Histamine	Injection	Some concerns	Continuous	Attack frequency
Monfared et al. [26]	2017	Melatonin	Oral	Low	Continuous	Attack frequency
Mulleners et al. [77]	2020	Galcanezumab	Injection	Low	Continuous, Dichotomous	MHDs
Noruzzadeh et al. [78]	2016	Memantine hydrochloride	Oral	Low	Continuous	Attacks per month
Ozyalcin et al. [32]	2005	Venlafaxine	Oral	High	Continuous	Headache days
Peikart et al. [79]	1996	Magnesium	Oral	High	Continuous, Dichotomous	Migraine days, Attack frequency
Petri et al. [80]	2009	Botox A	Injection	Low	Continuous	Attacks per month
Pfaffenrath et al. [81]	2002	MIG-99	Oral	Low	Continuous, Dichotomous	Attacks per month, Attack frequency
Pfaffernath et al. [82]	1996	Magnesium	Oral	High	Dichotomous	Intensity and duration
Pradalier et al. [83]	2001	Magnesium	Oral	Some concerns	Continuous	Attacks per month
Relja et al.^a^ [27]	2007	Botox A	Injection	Low	Continuous, Dichotomous	Episodes per month, Migraine episodes per month
Reuter et al. [84]	2018	Erenumab	Injection	Low	Continuous, Dichotomous	MMDs
Sadeghian et al. [85]	2015	Levetiracetam	Oral	High	Dichotomous	Headache attack frequency
Sakai et al. [86]	2020	Galcanezumab	Injection	Low	Continuous, Dichotomous	MHDs
Sakai et al. [87]	2021	Fremanezumab	Injection	Low	Continuous, Dichotomous	MMDs, Headache Days
Sakai et al. [88]	2021	Fremanezumab	Injection	Low	Continuous, Dichotomous	MMDs
Sakai et al. [89]	2019	Erenumab	Injection	Some concerns	Continuous, Dichotomous	MMDs
Sandor et al. [90]	2005	CoQ10	Oral	Low	Dichotomous	Attack frequency
Schoenen et al. [91]	1998	Riboflavin	Oral	Low	Continuous, Dichotomous	Attacks per month, Attack frequency
Silberstein et al. [92]	2020	Eptinezumab	IV	Low	Continuous, Dichotomous	MMDs
Silberstein et al. [93]	2007	Topiramate	Oral	Some concerns	Continuous	MMDs
Silberstein et al. [94]	2006	Topiramate	Oral	Some concerns	Continuous, Dichotomous	MMDs
Silbertstein et al. [95]	2017	Fremanezumab	Injection	Low	Continuous, Dichotomous	MMDs, Headache days
Siniatchkin et al. [96]	1998	Cyclandelate	Oral	High	Continuous	MMDs
Siniatchkin et al. [97]	2007	Metoprolol-CR	Oral	High	Continuous	Attacks per month
Skljarevski et al. [98]	2018	Galcanezumab	Injection	Low	Continuous, Dichotomous	MMDs, MHDs
Skljarevski et al. [99]	2018	Galcanezumab	Injection	Low	Continuous, Dichotomous	MHDs
Smith et al. [28]	2020	Eptinezumab	IV	Low	Continuous, Dichotomous	MMDs
Stauffer et al. [100]	2018	Galcanezumab	Injection	Low	Continuous, Dichotomous	MHDs
Steiner et al. [30]	1998	S-fluoxetine	Oral	High	Continuous	Attacks per month
Storey et al. [101]	2001	Topiramate	Oral	Some concerns	Continuous, Dichotomous	MMDs
Sun et al. [102]	2016	Erenumab	Injection	Low	Continuous, Dichotomous	MMDs
Takeshima et al. [103]	2021	Erenumab	Injection	Low	Continuous, Dichotomous	MMDs
Tepper et al. [104]	2017	Erenumab	Injection	Low	Continuous, Dichotomous	MMDs
Trapani et al. [105]	2000	Gabapentin	Oral	High	Continuous	Attack frequency
Vahedi et al. [106]	2002	Acetazolamide	Oral	High	Continuous	Attacks per month
Wang et al. [107]	2021	Erenumab	Injection	Low	Continuous, Dichotomous	MMDs

^a^Dichotomous outcome only used in sensitivity analysis

^b^Two different studies reported in same article

*Botox* Botulinum toxin, *IV* Intravenous, *MHD* Monthly headache day, *MMD* Monthly migraine day

### Results for continuous outcomes

The analysis of continuous outcomes consisted of 73 studies that enrolled 9,869 patients in the placebo arm. The median number of monthly migraine days at baseline was 8. The median [25th, 75th percentile] age of the patients was 41.0 [38.6, 42.3] years, and 85.9% of the patients were female. The median study duration was 13 [12, 17] weeks (Table 2). The mean study duration was not significantly different over the years with mean (SD) changing from 15.5 (4.0) weeks in the 1990s to 18.2 (11.3) by 2021 (Supplemental Table 1). However, sample size of trials has increased significantly (Supplemental Fig. 1). Additionally, the recent studies tended to have a higher proportion of female patients (77.9% during 1996–2004 vs. 88.0% during 2020–2021), permit the concurrent use of additional preventive migraine medication (15.4% during 1996–2004 vs. 58.3% during 2020–2021), and have a low risk of bias (38.5% during 1996–2004 vs. 100.0% during 2020–2021) (Supplemental Table 1). Studies with injectable and IV placebos became more common in recent years (Supplemental Fig. 2). There was a statistically significant difference in the mean change from baseline in the placebo arm by route of administration, with studies having an IV route showing greater overall placebo response and higher placebo response compared with injections. Mean (SD) change for IV was 4.7 (1.2), injections was 2.2 (1.5) for continuous outcomes (Table 3).

**Table 2:** Characteristics of studies reporting continuous outcomes (*n* = 73)

Variable	Median [25^th^, 75^th^ percentile]/ n (%)
Sample size of patients	89 [29, 222]
Study duration (weeks)	13 [12, 17]
Median improvement from baseline in the placebo arm	1.8 [1.0, 3.0]
Age	41.0 [38.6, 42.3]
% Female	85.9% [81.6%, 88.8%]
Placebo route	
*Oral*	35 (48.0%)
*Injection*	33 (45.2%)
*Intravenous*	5 (6.85%)
Year of publication	
*1996 to 2004*	13 (17.8%)
*2005 to 2009*	17 (23.3%)
*2010 to 2014*	7 (9.6%)
*2015 to 2019*	24 (32.9%)
*2020 to 2021*	12 (16.4%)
Outcome measure	
*Monthly migraine days*	33 (45.2%)
*Migraine headache days per month*	8 (1.0%)
*Attacks per month*	15 (21%)
*Episodes per month*	3 (4.1%)
*Migraine days*	7 (9.6%)
*Attack frequency*	5 (6.9%)
*Headache days per month*	0 (0.0%)
*Headache days*	2 (2.8%)
Jadad or Oxford scoring system	
*High*	71 (97.3%)
*Low*	2 (2.7%)
Cochrane risk of bias	
*High*	11 (15.1%)
*Some concerns*	14 (19.2%)
*Low*	48 (65.8%)
Use of migraine prophylaxis medication	
* Yes*	27 (37.0%)
* No*	40 (57.8%)
* Not reported*	6 (8.2)
Acute medication use permitted	
*Yes*	69 (94.5%)
*Not reported*	4 (5.5%)

**Fig. 2:**
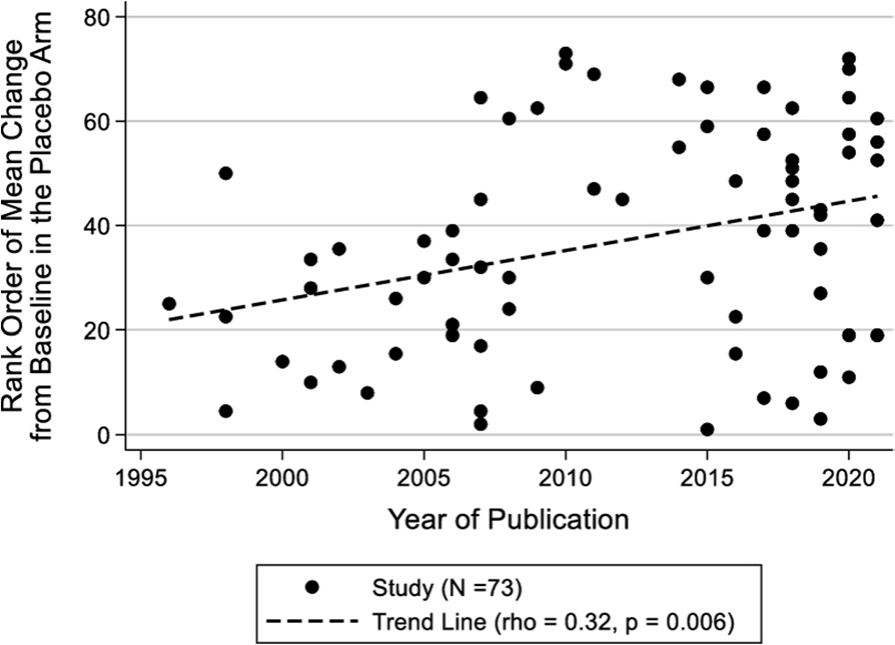
Change from baseline between mean change in the placebo arm and year of publication

**Table 3:** Mean change from baseline by route of administration

**Placebo Route**	**Mean (SD) Change from Baseline for Continuous Outcomes**	***P*-value**
Oral (*n* = 35)	1.5 (1.5)	
Injection (*n* = 33)	2.2 (1.5)	< 0.001
Intravenous (*n* = 5)	4.7 (1.2)	
**Placebo Route**	**Mean (SD) Change from Baseline for Dichotomous Outcomes**	***P*-value**
Oral (*n* = 23)	26.7% (15.9)	
Injection (*n* = 21)	24.5% (14.0)	0.011
Intravenous (*n* = 6)	45.1% (7.8)	

*Abbreviations: SD* Standard deviation

#### Correlation analysis of placebo response with publication year

The rank order (the lowest value is ranked 1, the second lowest is ranked 2, etc.) of the placebo mean change from baseline is plotted against publication year in Fig. 2. There was a positive correlation, indicating an increase in placebo response over the years (rho = 0.32, *p* = 0.006). Sensitivity analysis by route of administration (oral studies and oral/injectable studies) showed similar values (Supplemental Table 2).

#### Regression analysis of continuous outcomes

**Fig. 3:**
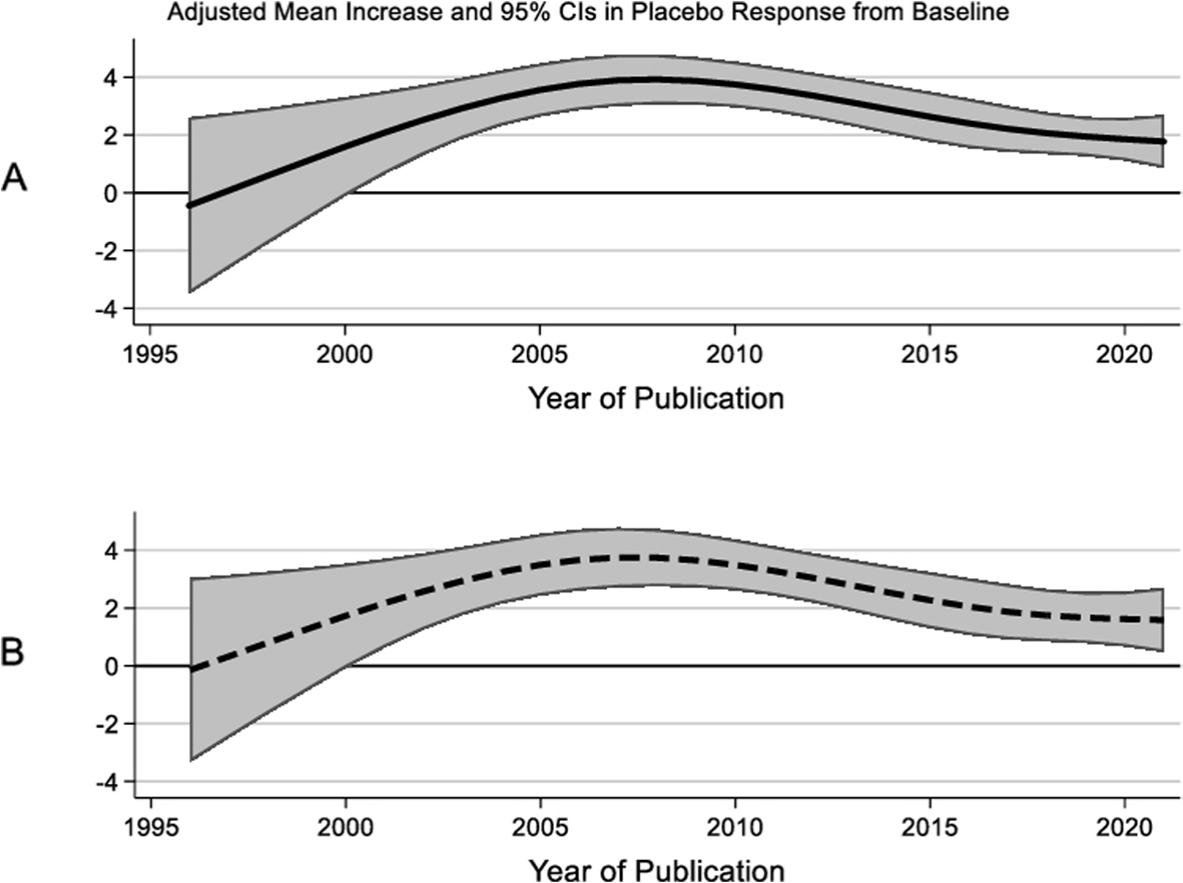
Mean change in placebo response **A**) adjusted for patient age, sex, route of administration, Cochrane risk of bias, and year of publication and **B**) adjusted for study duration, type of efficacy outcome, permission to use other prophylaxis medications along with predictors listed in ‘A’

Due to missing covariate data, 71 out of the 73 included studies were available for regression modeling. The final regression model adjusted for each study’s mean reported age, percent female, route of administration, Cochrane RoB, and year of publication, with each year modeled as a restricted cubic spline with 4 knots. The regression model also weighted the results by sample size of patients in the study. A significant, positive, but non-linear trend between year of publication and placebo response was observed (Fig. 3A). Notably, studies with older patients in the placebo arm had a lower placebo response.

While IV administration was associated with significantly higher placebo response compared to injectable administration, injectable and oral placebos did not differ after multivariable adjustment (Table 4). As an initial sensitivity analysis, we added the variables representing study duration, type of efficacy outcome, and permission to use additional preventive medications, to the original model. No significant change was observed in the relationship between year of publication and placebo response after adjusting for these additional variables (Fig. 3B). Next, as a second sensitivity analysis, studies with “high risk of bias” followed by “high risk or some concerns of bias” were excluded from the model, which also produced similar results as the final regression model (Supplemental Fig. 3), indicating that studies with a high risk or some concerns of bias did not confound the relationship between placebo response and publication year. Additionally, we performed a sensitivity analysis on the studies that had no imputation, and observed a very similar relationship with year of publication and outcome (each spline term for year of publication was similarly statistically significant).

**Table 4:** Regression model for continuous outcomes (71 studies)

Variable	Coefficient	95% CI	*P*-value
Constant	-1021.7	-1783.5 to -259.9	0.009
Year (Spline Term #1) per year	0.5	0.1 to 0.9	0.009
Year (Spline Term #2) per year	-1.0	-1.7 to -0.3	0.008
Year (Spline Term #3) per year	2.2	0.2 to 4.3	0.033
% Female (per .01 increase)	0.1	-0.0 to 0.1	0.176
Mean age of patients (per year)	-0.2	-0.3 to -0.0	0.043
Route of administration			
*Injection (reference)*	0		
*Intravenous*	2.3	1.2 to 3.5	< 0.001
*Oral*	-0.3	-1.4 to 0.8	0.619
Cochrane risk of bias			
*Low risk (reference)*	0		
*Some concerns*	-2.0	-3.3 to -0.7	0.003
*High risk*	-1.0	-3.4 to 1.5	0.428

The spline terms #2 and #3 can be interpreted as changes to the overall slope (presenting as Spline Term #1) at different intervals of year. See Fig. 3 for a visual display of the relationship between Year of Publication and Placebo Response. For the variable Mean Age, the model shows that for every increase of 1 year, the Placebo Response (the difference between the placebo outcome from baseline) decreases by 0.2 units. For Route of Administration, placebos administered intravenously had a mean placebo response that was 2.3 units higher compared to the referent category of Injection route

*CI* Confidence Interval

### Results for dichotomous outcomes

**Fig. 4:**
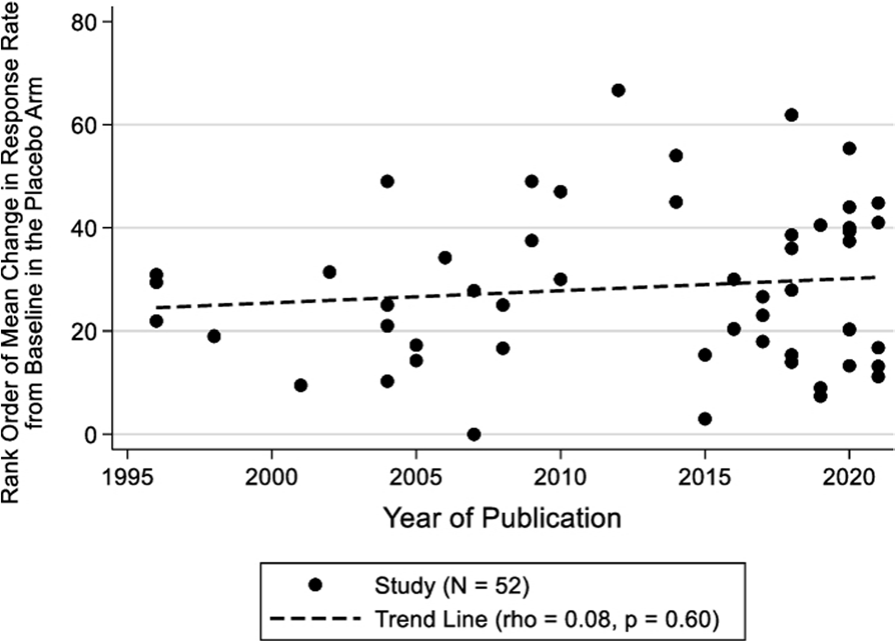
Responder rate in the placebo arm (percent of patients with ≥ 50% reduction in MMDs, MHDs, attack frequency, migraine episodes per month, migraine days, headache attack frequency, headache days, and intensity or duration) versus year of publication

The analysis of dichotomous outcomes consisted of 52 studies that enrolled 7,913 patients in the placebo arm (Table 5). The median [25th, 75th percentile] age of the patients was 41.1 [39.0, 42.2] years, and 86.2% of the patients were female. The rank order of the mean change from baseline in the placebo arm is plotted against the publication year in Fig. 4. There was no significant correlation between the percent responder rate and year of publication, indicating a lack of a linear trend (rho = 0.08, *p* = 0.596). Sensitivity analysis by route of administration produced similar results. In the regression analysis of the 52 studies reporting dichotomous responses there was no significant relationship between year of publication and mean placebo response (Table 6). Similar to the continuous outcomes, we performed a sensitivity analysis on the studies with no imputation, and observed a very similar relationship with year of publication and outcome.

**Table 5:** Characteristics of all studies reporting dichotomous outcomes (*n* = 52)

Variable	Median [25^th^, 75^th^ Percentile]
Sample Size	113 [38, 230]
Study Duration (weeks)	13 [12, 17]
Age	41.1 [39.0, 42.2]
% Female	86.2% [81.6%, 88.5%]
Median Improvement from Baseline in the Placebo Arm	27.2 [16.1, 39.7]
Placebo Route	N (%)
*Oral*	23 (42.3%)
*Injection*	22 (40.4%)
*Intravenous*	6 (11.5%)
*Unknown*	2 (5.8%)
Cochrane Risk of Bias	
*Low*	39 (75.0%)
*Some Concerns*	8 (15.4%)
*High*	5 (9.6%)
Year of Publication	
*1996 to 2004*	10 (19.2%)
*2005 to 2009*	9 (17.3%)
*2010 to 2014*	5 (9.6%)
*2015 to 2019*	16 (30.8%)
*2020 to 2021*	12 (23.1%)
Outcome Measure (% responder rate)	
*Monthly migraine days*	26 (50.0%)
*migraine headache days*	11 (21.2%)
*attack frequency*	8 (15.4%)
*migraine episodes per month*	0 (0.0%)
*migraine days*	3 (5.8%)
*headache attack frequency*	1 (1.9%)
*headache days*	2 (3.9%)
*intensity and duration*	1 (1.9%)

**Table 6:** Regression model for dichotomous outcomes (50 studies)

Covariate	Coefficient	95% Confidence Interval	*P*-value
Constant	-244.1	-2103.0 to 1614.9	0.792
Year (per year)	0.2	-0.7 to 1.1	0.682
Age (per year)	-2.4	-4.0 to -0.9	0.003
% Female (per 0.01 increase)	-0.1	-1.0 to 0.7	0.777
Cochrane Level of Bias			
*Low (reference)*	0		
*Some*	-9.8	-25.3 to 5.8	0.213
*High*	-11.0	-35.9 to 13.9	0.377
Route			
*Injection (reference)*	0		
*Oral*	6.4	-4.8 to 17.6	0.253
*IV*	11.5	1.5 to 21.4	0.025

*Abbreviations: IV* Intravenous

## Discussion

These analyses found a statistically significant increase in the placebo response in preventive migraine studies from 1990 to 2021 when examining continuous efficacy outcomes. This was observed in the correlation analysis and a series of multivariable regression models. The regression results should be considered more definitive than the correlations given that they are adjusted for the characteristics of each study and the sample size. We also observed that the placebo response significantly varied by route (oral, injectable, and IV), a finding supported in the literature in smaller meta-analyses of migraine prevention studies [13, 108–110]. Moreover, this comprehensive literature review showed that preventive migraine trials have changed over the years. The trials have become larger, are reporting different efficacy outcomes, and are less likely to examine oral medications.

The current results warrant comment in relation to Evans et al*.* who evaluated the relationship between the degree of placebo response and the difference between drug and placebo in 28 migraine prophylaxis trials in a meta-analysis over a time period similar to our study [13]. Evans et al*.* only examined the proportion of ≥ 50% responders, defined as a ≥ 50% reduction in migraine days/month. After adjusting for a similar set of confounders as this study, they did not find a significant linear relationship between year of publication in placebo response [13]. In our larger analysis of 52 studies incorporating a more diverse group of *dichotomous* outcomes, we also did not find a relationship with year of publication and placebo response. However, when we examined 71 *continuous* outcome studies and permitted the year to have a non-linear relationship with the placebo response, we found a significant, positive association between year of publication and placebo response. While we found that dichotomous outcomes may not show as much sensitivity to placebo response over the past 30 years compared to continuous outcomes due to low granularity, they may be considered reliable clinical endpoints since they revealed a constant placebo response over the years in this analysis.

In general, more invasive treatments yield higher placebo responses [108]. Our findings confirm this, as we observed placebos administered IV had a significantly greater placebo response than either injectable or oral placebos when including all of the acceptable prespecified continuous outcome measures. Injectable and oral placebos had similar placebo responses after multivariable adjustment, though injectable placebos had a significantly greater unadjusted placebo response than oral placebos among studies with continuous efficacy outcomes. Meissner et al*.* found that invasive interventions, such as sham acupuncture and sham surgery had higher responder ratios than oral placebos in a meta-analysis of migraine prophylaxis studies, and, similar to our study, also found sham (placebo) injections had similar responses as oral placebos [15]. A meta-analysis by Swerts et al*.* found placebo responses to oral topiramate and subcutaneous monoclonal antibodies were not statistically different, but were inferior to head injection or IV infusion [108]. In contrast, de Craen et al*.*, in an older meta-analysis of acute migraine treatment trials of sumatriptan, showed a 32.4% response with subcutaneous placebos versus 25.7% with oral placebos [109]. Potential explanations for invasive treatments yielding a higher placebo response in our study include contextual effects due to patient perceptions, medication cost, previous expectations with pain medications, and newness of intervention. Patients consider invasive treatments as ‘‘stronger’’ or more efficient [111]. Higher cost of medications have shown to produce better pain relief compared to discounted medication [112]. Placebo response is typically greater for newer treatments. New medications show a larger placebo response than those established, suggesting enhanced patient expectations with ‘modern’ medications [113]. It is rather unexpected that the discovery of new migraine prophylaxis treatment in recent years such as CGRP inhibitors did not lead to a sharp increase in placebo effect in our analysis. Patient's expectations can influence the placebo effect as they may have heard about the development of an innovative treatment (e.g., through media) and may believe that it will be effective. However, with passing time and experience with therapy, the strong initial expectations subside [114]. Other factors, such as mode of administration and clinical environment, all contribute to the “contextual effect” [115]. Understanding and controlling for these factors is essential in designing and interpreting clinical trials for migraine prevention treatments. More investigation is needed to explore contextual effects, which may provide an opportunity to adjust the impact of placebo as well as enhance health outcomes by focusing on patient expectations, interaction with provider, setting, and culture.

It is beyond our study’s scope to determine why the placebo response was associated with an increase over the past 30 years even after adjusting for route of administration and other potential confounders. However, “unspecific effects” of both increased clinical attention and expectation bias may have played a role. Placebo administration is effectively a non-specific treatment where the intensive contact with the clinical staff can produce symptom improvement, particularly in less severely ill participants [116, 117]. Moreover, patients with more severe symptoms may have had increased expectations of symptom relief over the years. Sanders et al*.* found that greater pain sensitivity may manifest as greater expectations of and need for pain relief [118]. Heightened expectations tend to nullify differences between treatment and placebo analgesia [118]. Since patient-reported outcomes are subjective especially in conditions such as pain, placebo response is less avoidable compared to biomarker-based outcomes [11]. Also, as previously stated, increased placebo responses over the past 30 years is a phenomenon observed in other therapeutic areas, which warrants further study.

From our multivariable analyses, we observed a steady increase in placebo response up until the mid 2000s, when the association thereafter essentially plateaued. This non-linear relationship could be an artefact of the data or could truly reflect real changes in the studies known to have happened during this time [119, 120]. Our systematic literature review shows that older studies tended to use outcomes such as “migraine attacks/episodes” which are infrequently reported in the past decade. In 2008, the International Headache Society (IHS) Clinical Trials Standing Committee published the first edition of the *Guidelines for controlled trials of prophylactic treatment of chronic migraine in adults* to assist in the design of well-controlled clinical trials [120]. Some recommendations were 1) to include a baseline observation period of at least 1 month, 2) to ensure a treatment period of at least 3 months, 3) question subjects and investigators at the end of the trial regarding their opinion as to what treatment group (active or placebo) the subject was assigned to during the study, 4) randomization of subjects in relatively small blocks, and 5) regular follow-up of subjects to determine eligibility, ensure compliance, and monitor for adverse events; all of which could decrease placebo response. Therefore, the reduction in placebo response after 2008 could be due to implementation of these guidelines. Placebo response may have also stabilized in recent years because of improved symptom reporting by patients using electronic diaries. Research suggests patients who are not proficient at discerning and reporting their own symptoms demonstrate a high placebo response attributable to inaccurate reporting of symptom intensity [13].

The goal of all therapeutic trials should be to minimize the placebo effect in clinical trials, while maximizing its effect in clinical practice [121]. Newer clinical trial designs may better control placebo response. For example, a sequential parallel comparative design study (SPCD) conducted in two phases may eliminate high placebo responders. In the first phase, the randomization between the active treatment and placebo are unbalanced in favor of placebo. In the second phase, placebo non-responders are re-randomized to either active treatment or placebo. At the end of the trial, data from both phases are pooled for final analysis. The drawback, however, is that it represents a more complex and time intensive study design [121, 122]. A placebo run-in phase is another method to minimize the placebo response, but care should be taken when implementing this solution, as it requires a larger sample size, an additional study phase, and its clinical usefulness has been debated [13, 121]. Another solution would be training to improve symptom reporting [13]. Patient expectation bias can be minimized through training, such as Placebo Response Reduction Training [13]. Considering that sophisticated structures of RCTs may not accurately reflect real-world clinical needs, particularly in chronic conditions, where the effects of treatment may take longer to manifest, it is important to develop solutions that evolve beyond meta-analyses and systematic reviews. The narrowing of the gap between active substance and placebo further necessitates the focus on developing innovative techniques that are better suited to evaluating the effectiveness of different treatments. In context of healthcare research, quantum computing may offer new opportunities to identify patterns and relationships that are not readily apparent with traditional methods and address the issue of placebo response [123, 124]. In situations where direct comparisons and network meta-analyses are not feasible, alternative methods such as unanchored simulated treatment comparisons can be utilized for synthesizing evidence on comparative effectiveness and help accelerate the development of new and more effective treatments for a range of diseases [125].

The clinical implications of an increasing placebo response over the years in migraine RCTs are both subtle and profound. An increased placebo response leads to decreased efficacy differences between placebo and treatment, and so larger sample sizes are needed when designing a clinical trial [126]. This ultimately increases RCT cost and time to completion [127, 128]. Also, if power calculations are based on results from older studies, an RCT may be inadvertently underpowered for a contemporary population. Additionally, increasing temporal trends in placebo response may bias future meta-analyses intended to compare the effectiveness of preventive migraine medications when the time span of included trials is large, as the same treatment may appear “better” among older studies. Hence, further characterization of placebo response based on study characteristics is warranted. Placebo response also has implications in clinical practice. Considering ethical approaches, physicians can enhance and strengthen the effectiveness of medical treatments by using placebo as an additive effect or supplement to active treatment [129]. However, placebo medications should be used with caution, due to masking and potentially delaying the treatment of medical conditions [130]. Although our study focuses on pharmacological interventions in preventive migraine, patients receiving behavioral interventions such as relaxation training or cognitive behavioral therapy, and non-pharmacological interventions such as sham acupuncture or surgery may also experience placebo responses. It has been established that the context and meaning of a placebo therapy are more important than the placebo vehicle itself [131]. However, the context and meaning of different therapies for migraine treatment can differ considerably. For example, patients could have higher expectations from treatments such as acupuncture or surgery because of the more elaborate and impressive treatment rituals [132]. Placebo response can also be enhanced due to high expectations from newer treatments such as cognitive behavioral therapy [114]. More studies are warranted to study changing patient expectations and placebo response in non-pharmacological treatments, which may enhance treatment of migraine symptoms.

While our study’s strengths include the large number of clinical trials analyzed, the detailed data abstraction, and its advanced statistical methods, it does have some limitations. First, as with any literature review and meta-analysis, only articles published in English were included. Clinical trials failing to meet their primary efficacy endpoint may go unpublished [133], and so the true placebo response in migraine preventive trials may be underestimated. Although we adjusted for many predictors used in other studies such as study duration and route of administration, unmeasured confounding due to exclusion of pediatric patients, lack of diversity in sex, age, and lack of information on other demographics (e.g., race, ethnicity, socio-economic status) may bias the observed relationship between year of publications and placebo response. Consequently, a future study adjusting for additional study characteristics may produce different findings. Although the scope of our current study did not differentiate between the types of migraine (episodic or chronic with or without aura) a prior study found similar contextual effect in episodic, chronic, and combined population for IV and injectable treatments [115]. Finally, cross-over studies meeting the inclusion criteria often did not provide results for the first treatment phase and were thus excluded, which may have limited the data available for analysis in our study.

## Conclusions

In this review examining placebo response over the past 30 years in migraine prophylaxis, we found a statistically significant increase in the placebo response in preventive migraine trials from 1990 to 2021. The increase was not constant over the years or detectable when the outcome was reported as a dichotomous variable, which highlights the nuanced relationship between placebo response and year of publication. This study found that different sources of heterogeneity, such as route of administration and patient characteristics, affect placebo response. Meta-analyses and studies assessing therapeutic gain using the differential responses between treatment and placebo arms in migraine studies may be biased given the variable placebo response over the years observed in our findings. Hence, future studies should account for placebo response over the years in their analysis and clinical trials could incorporate placebo run-in periods, adjust for placebo response trends, and account for patient and study characteristics in their analyses.

## Supplementary Information

**Additional file 1.** Search terms used in the PubMed database.

**Additional file 2.** Search terms used in the EMBASE database.

**Additional file 3: Supplemental Table 1.** Select variables stratified by year of publication for studies reporting continuous outcomes. **Supplemental Table 2.** Sensitivity analysis of continuous outcomes by the route of administration. **Supplemental Table 3.** Cochrane risk-of-bias tool for randomized trials. **Supplemental Figure 1.** Mean study sample size over time for studies reporting continuous outcomes. **Supplemental Figure 2.** Placebo route over time among eligible studies reporting continuous outcomes. **Supplemental Figure 3.** Adjusted mean change in placebo response from baseline on studies with A) low risk and some concern of bias and B) only low risk of bias.

## Data Availability

The dataset analyzed during the current study are available from the corresponding author on reasonable request.

## Abbreviations

CGRP: Calcitonin Gene-Related Peptide
RCT: Randomized Controlled Trial
MA: Meta-Analysis
PICOS: Population, Intervention, Comparison, Outcomes, and Study
IV: Intravenous
MMD: Monthly Migraine Day
MHD: Monthly Headache Day
RoB: Risk of Bias
PRISMA: Preferred Reporting Items for Systematic Reviews and Meta-Analyses
Botox: Botulinum toxin
