# Supplementary material for: The temporal trend of placebo response in migraine prevention from 1990 to 2021: a systematic literature review and meta-analysis with regression
Source: J Headache Pain. 2023 May 16;24(1):54. doi: 10.1186/s10194-023-01587-0 (PMC10189936; doi:10.1186/s10194-023-01587-0)
Supplement: Supplementary file 1 — Additional file 1. Search terms used in the PubMed database. [file 10194_2023_1587_MOESM1_ESM.docx]

**Additional file 1. Search terms for Pubmed**

- The search terms used in Pubmed are displayed below: (08/15/21)

Search was filtered by year 1990 to 2021

{randomized controlled trial [pt] OR controlled clinical trial [pt] OR randomized controlled trials [mh] OR random allocation [mh] OR double-blind

method [mh] OR single-blind method [mh] OR clinical trial [pt] OR clinical trials [mh] OR (clinic* [tw] AND trial* [tw]) OR (singl* [tw] OR doubl* [tw] OR

trebl* [tw] OR tripl* [tw])} NOT (animal [mh] NOT human [mh])

AND placebo* [tw]

AND migrain* NOT (Stroke [TI] OR Cardiac [TI] OR Cardiovascular [TI] OR depression [TI] OR mania [TI] OR anxiety [TI] OR epilepsy [TI])

AND (sodium valproate OR Topiramate OR Carbamazepine OR Gabapentin OR Metoprolol OR Propranolol OR Timolol OR Atenolol OR Nebivolol

OR Pindolol OR Nadolol OR Bisoprolol OR Candesartan OR Lisinopril OR Guanfacine OR Acetazolamide OR Nicardipine OR Nifedipine OR

Nimodipine OR Flunarizine OR Verapamil OR Cyclandelate OR Clonidine OR Amitriptyline OR Protriptyline OR Venlafaxine OR Desvenlafaxine OR

Duloxetine OR Fluoxetine OR Fluvoxamine OR Naratriptan OR Fenoprofen OR Ibuprofen OR Ketoprofen OR Naproxen OR Flurbiprofen OR

Mefenamic acid OR Indomethacin OR Erenumab OR Fremanezumab OR Galcanezumab OR Eptinezumab OR Atogepant OR Rimegepant OR

Onabotulinumtoxin A OR Histamine OR Cyproheptadine OR Clonidine OR Acenocoumarol OR Coumadin OR Aspirin OR Picotamide OR Petasites

OR Feverfew OR Magnesium OR Riboflavin OR Omega-3 OR Coenzyme Q10 OR Pizotifen OR Methysergide OR Memantine OR Milnacipran OR

Phenelzine OR Tranylcypromine OR Isocarboxazid OR Methylergonovine OR methylergometrine OR Tizanidine)

AND (Prevent* OR Prophyla*)

- The same search terms were used in Cochrane Library (08/13/21)

Search was filtered by publication year 1990-2021 and Trials
