# Supplementary material for: The temporal trend of placebo response in migraine prevention from 1990 to 2021: a systematic literature review and meta-analysis with regression
Source: J Headache Pain. 2023 May 16;24(1):54. doi: 10.1186/s10194-023-01587-0 (PMC10189936; doi:10.1186/s10194-023-01587-0)
Supplement: Supplementary file 2 — Additional file 2. Search terms used in the EMBASE database. [file 10194_2023_1587_MOESM2_ESM.docx]

**Additional file 2. Search terms for EMBASE**

EMBASE used the following search terms and filter. (08/06/21)

‘randomized controlled trial’:it OR ‘controlled clinical trial’:it OR ‘randomized controlled trial’/exp OR randomization/exp OR ‘double blind procedure’/exp OR ‘single blind procedure’/exp OR ‘clinical trial’:it OR ‘clinical trial’/exp OR (clinic* AND trial*).mp. OR (singl* OR doubl* OR trebl* OR tripl*).mp.

NOT

'animal experiment' NOT 'human experiment'

AND

placebo*.mp.

AND

migrain*

NOT

Stroke.ti. OR Cardiac.ti. OR Cardiovascular.ti. OR depression.ti. OR mania.ti. OR anxiety.ti. OR epilepsy.ti.

AND

Sodium valproate OR Topiramate OR Carbamazepine OR Gabapentin OR Metoprolol OR Propranolol OR Timolol OR Atenolol OR Nebivolol OR Pindolol OR Nadolol OR Bisoprolol OR Candesartan OR Lisinopril OR Guanfacine OR Acetazolamide OR Nicardipine OR Nifedipine OR Nimodipine OR Flunarizine OR Verapamil OR Cyclandelate OR Clonidine OR Amitriptyline OR Protriptyline OR Venlafaxine OR Desvenlafaxine OR Duloxetine OR Fluoxetine OR Fluvoxamine OR Naratriptan OR Fenoprofen OR Ibuprofen OR Ketoprofen OR Naproxen OR Flurbiprofen OR Mefenamic acid OR Indomethacin OR Erenumab OR Fremanezumab OR Galcanezumab OR Eptinezumab OR Atogepant OR Rimegepant OR Onabotulinumtoxin A OR Histamine OR Cyproheptadine OR Clonidine OR Acenocoumarol OR Coumadin OR Aspirin OR Picotamide OR Petasites OR Feverfew OR Magnesium OR Riboflavin OR Omega-3 OR Coenzyme Q10 OR Pizotifen OR Methysergide OR Memantine OR Milnacipran OR Phenelzine OR Tranylcypromine OR Isocarboxazid OR Methylergonovine OR methylergometrine OR Tizanidine

AND

Prevent* OR Prophyla*

This database was filtered to publication year 1990-present
