# Supplementary material for: The temporal trend of placebo response in migraine prevention from 1990 to 2021: a systematic literature review and meta-analysis with regression
Source: J Headache Pain. 2023 May 16;24(1):54. doi: 10.1186/s10194-023-01587-0 (PMC10189936; doi:10.1186/s10194-023-01587-0)
Supplement: Supplementary file 3 — Additional file 3: Supplemental Table 1. Select variables stratified by year of publication for studies reporting continuous outcomes. Supplemental Table 2. Sensitivity analysis of continuous outcomes by the route of administration. Supplemental Table 3. Cochrane risk-of-bias tool for randomized trials. Supplemental Figure 1. Mean study sample size over time for studies reporting continuous outcomes. Supplemental Figure 2. Placebo route over time among eligible studies reporting continuous outcomes. Supplemental Figure 3. Adjusted mean change in placebo response from baseline on studies with A) low risk and some concern of bias and B) only low risk of bias. [file 10194_2023_1587_MOESM3_ESM.docx]

**Supplemental data**

**Supplemental Table 1. Select variables stratified by year of publication for studies reporting continuous outcomes (n=73)**

| **Variable** | **Year** | | | | | **P-value** |
| --- | --- | --- | --- | --- | --- | --- |
|  | 1996-2004  n=13 | 2005-2009  n=17 | 2010-2014  n=7 | 2015-2019  n=24 | 2020-2021  n=12 |  |
| Age (Mean, SD) | 40.0 (3.8) | 41.1 (3.4) | 40.1 (2.4) | 39.3 (4.6) | 41.4 (2.4) | 0.397 |
| Mean % Female (SD) | 77.9% (14.3) | 87.4% (7.5) | 85.9% (5.9) | 83.2% (7.9) | 88.0% (4.4) | 0.018 |
| Use of Migraine Prophylaxis Medication |  |  |  |  |  |  |
| *Yes (%)* | 15.4% | 29.4% | 28.6% | 45.8% | 58.3% | 0.027 |
| Cochrane Risk of Bias |  |  |  |  |  |  |
| *Low (%)* | 38.5% | 35.3% | 85.7% | 79.2% | 100.0% | <0.001 |
| Study Duration in Weeks (Mean, SD) | 15.5 (4.0) | 14.9 (5.1) | 16.9 (7.5) | 13.8 (6.5) | 18.2 (11.3) | 0.474 |

Abbreviations: n, number of studies; SD, standard deviation.

**Supplemental Table 2. Sensitivity analysis of continuous outcomes by the route of administration (correlation between year and ranking of mean reduction from baseline)**

| **Correlation Description** | **Rho; P-value** |
| --- | --- |
| Spearman’s rank correlation on all eligible studies (n=73) | 0.32; p= 0.006 |
| **Sensitivity Analyses** | **Rho; P-value** |
| Spearman’s rank correlation on oral placebo studies (n=35) | 0.29; p = 0.090 |
| Spearman’s rank correlation on injectable placebo studies (n=33) | -0.01; p = 0.947 |
| Spearman’s rank correlation on injectable and oral placebo studies but not IV studies (n=68) | 0.25; p= 0.039 |

Abbreviations: n, number of studies

**Supplemental Table 3. Cochrane risk-of-bias tool for randomized trials**

| **Domain 1: Risk of bias arising from the randomization process** | **Response options** |
| --- | --- |
| 1.1 Was the allocation sequence random? | Y / PY / PN / N / NI |
| 1.2 Was the allocation sequence concealed until participants were enrolled and assigned to interventions? | Y / PY / PN / N / NI |
| 1.3 Did baseline differences between intervention groups suggest a problem with the randomization process? | Y / PY / PN / N / NI |
| **Domain 2: Risk of bias due to deviations from the intended interventions** *(effect of assignment to intervention)* | **Response options** |
| 2.1. Were participants aware of their assigned intervention during the trial? | Y / PY / PN / N / NI |
| 2.2. Were carers and people delivering the interventions aware of participants' assigned intervention during the trial? | Y / PY / PN / N / NI |
| 2.3. If Y/PY/NI to 2.1 or 2.2: Were there deviations from the intended intervention that arose because of the trial context? | NA / Y / PY / PN / N / NI |
| 2.4 If Y/PY to 2.3: Were these deviations likely to have affected the outcome? | NA / Y / PY / PN / N / NI |
| 2.5. If Y/PY/NI to 2.4: Were these deviations from intended intervention balanced between groups? | NA / Y / PY / PN / N / NI |
| 2.6 Was an appropriate analysis used to estimate the effect of assignment to intervention? | Y / PY / PN / N / NI |
| 2.7 If N/PN/NI to 2.6: Was there potential for a substantial impact (on the result) of the failure to analyse participants in the group to which they were randomized? | NA / Y / PY / PN / N / NI |
| **Domain 2: Risk of bias due to deviations from the intended interventions** *(effect of adhering to intervention)* | **Response options** |
| 2.1. Were participants aware of their assigned intervention during the trial? | Y / PY / PN / N / NI |
| 2.2. Were carers and people delivering the interventions aware of participants' assigned intervention during the trial? | Y / PY / PN / N / NI |
| 2.3. [If applicable:] If Y/PY/NI to 2.1 or 2.2: Were important non-protocol interventions balanced across intervention groups? | NA / Y / PY / PN / N / NI |
| 2.4. [If applicable:] Were there failures in implementing the intervention that could have affected the outcome? | NA / Y / PY / PN / N / NI |
| 2.5. [If applicable:] Was there non-adherence to the assigned intervention regimen that could have affected participants’ outcomes? | NA / Y / PY / PN / N / NI |
| 2.6. If N/PN/NI to 2.3, or Y/PY/NI to 2.4 or 2.5: Was an appropriate analysis used to estimate the effect of adhering to the intervention? | NA / Y / PY / PN / N / NI |
| **Domain 3: Missing outcome data** | **Response options** |
| 3.1 Were data for this outcome available for all, or nearly all, participants randomized? | Y / PY / PN / N / NI |
| 3.2 If N/PN/NI to 3.1: Is there evidence that the result was not biased by missing outcome data? | NA / Y / PY / PN / N |
| 3.3 If N/PN to 3.2: Could missingness in the outcome depend on its true value? | NA / Y / PY / PN / N / NI |
| 3.4 If Y/PY/NI to 3.3: Is it likely that missingness in the outcome depended on its true value? | NA / Y / PY / PN / N / NI |
| **Domain 4: Risk of bias in measurement of the outcome** | **Response options** |
| 4.1 Was the method of measuring the outcome inappropriate? | Y / PY / PN / N / NI |
| 4.2 Could measurement or ascertainment of the outcome have differed between intervention groups? | Y / PY / PN / N / NI |
| 4.3 If N/PN/NI to 4.1 and 4.2: Were outcome assessors aware of the intervention received by study participants? | NA / Y / PY / PN / N / NI |
| 4.4 If Y/PY/NI to 4.3: Could assessment of the outcome have been influenced by knowledge of intervention received? | NA / Y / PY / PN / N / NI |
| 4.5 If Y/PY/NI to 4.4: Is it likely that assessment of the outcome was influenced by knowledge of intervention received? | NA / Y / PY / PN / N / NI |
| **Domain 5: Risk of bias in selection of the reported result** | **Response options** |
| 5.1 Were the data that produced this result analysed in accordance with a pre-specified analysis plan that was finalized before unblinded outcome data were available for analysis? | Y / PY / PN / N / NI |
| Is the numerical result being assessed likely to have been selected, on the basis of the results, from... |  |
| 5.2. ... multiple eligible outcome measurements (e.g. scales, definitions, time points) within the outcome domain? | Y / PY / PN / N / NI |
| 5.3 ... multiple eligible analyses of the data? | Y / PY / PN / N / NI |
| Abbreviations: N, no; NA, not applicable; NI, no information; PN, probably no; PY, probably yes; Y, yes | |

**Note:** Responses to questions within each domain are mapped onto a proposed risk-of-bias judgement, which include the following options: (1) low risk of bias; (2) some concerns; and (3) high risk of bias. These domain-level judgements in turn, provide the same basis for an overall risk-of-bias judgement across domains for the outcome. An overall judgement of low risk of bias indicates that the study is judged to be at low risk of bias for all domains; some concerns indicate that the study is judged to raise some concerns in at least one domain, but not to be at high risk of bias for any domain; and finally, high risk of bias indicates the study is judged to be at high risk of bias in at least one domain, or has some concerns for multiple domains in a way that substantially lowers confidence in the result.

**Supplemental Figure 1. Mean study sample size over time for studies reporting continuous outcomes (n=73)**

**Supplemental Figure 2. Placebo route over time among eligible studies reporting continuous outcomes (n=73)**

**Supplemental Figure 3. Adjusted mean change in placebo response from baseline on studies with A) low risk and some concern of bias and B) only low risk of bias**
